# 573 Feasibility of an Unfunded Multicenter Trial Group for Older Adult Burn Patients: A Retrospective Study

**DOI:** 10.1093/jbcr/iraf019.202

**Published:** 2025-04-01

**Authors:** Lauren Nosanov, Alisa Savetamal, Jessica Reynolds, Dhaval Bhavsar, Sam Miotke, Mark Johnston, Lucy Wibbenmeyer, Colette Galet, David Hill, Sara Higginson, Theresa Chin, Andrea Long, Emily Baker, Hannah Hamm, Alexandra Lacey, Rosemary Paine, Diana Tedesco, Shawn Tejiram, Marc Jeschke, Kathleen Romanowski

**Affiliations:** Emory University School of Medicine; Connecticut Burn Center, Bridgeport Hospital / Yale-New Haven Health; The University of Kansas Health System; The University of Kansas Health System Burnett Burn Center; Regions Hospital; Regions Hospital; University of Iowa; University of Iowa; Regional One Health; Shriners Children’s - Ohio; University of California Irvine; University of California San Francisco Fresno; Hamilton Health Sciences; Hamilton Health Sciences; Regions Hospital Burn Center; Maine Medical Center; Hamilton Health Sciences; MedStar Washington Hospital Center; HHS and McMaster University; University of California, Davis and Shriners Children’s

## Abstract

**Introduction:**

Older adults represent a significant portion of the burn patient population, presenting unique physiological challenges and requiring tailored treatment approaches. Yet, comprehensive data specific to this demographic are limited. The relatively small number of older burn patients at each center makes single-center studies insufficient to address key questions about their care. The development of a multicenter trial group focused on older adult burn patients could yield valuable insights into clinical outcomes, yet funding for such initiatives is often restricted. This study explores the feasibility of creating an unfunded multicenter trial group and conducting a retrospective pilot study to examine clinical trends and outcomes for older adult burn patients.

**Methods:**

Following IRB approval and the creation of data use agreements, a retrospective pilot study was conducted across twelve burn centers in North America. Each center was tasked with collecting standardized data on burn patients aged 60 and older from January 2017 to December 2019. Data included demographic information, burn characteristics (total body surface area burned, type of burn, etc.), treatment interventions, and outcomes such as mortality, length of hospital stay, and complications. Where possible, data were sourced from each center’s submission to the Burn Care Quality Platform (BCQP). BCQP and additional data were manually entered into a centralized REDCap database managed by one institution. Descriptive statistics of the pilot study are presented.

**Results:**

The study included 1,632 older adult burn patients. Median age was 68 years (interquartile range [IQR] 13); 1,095 patients (67%) were male and 1,187 (73%) were White with a median BMI of 27.5 (IQR 7.87). The median burn size was 3.5% total body surface area (IQR 9, range 0-100%). Patients presented a median of 4.68 hours after injury (IQR 15.35), with a median modified Baux score of 76.21 (IQR 20.5). These patients underwent a median of 1 operation (IQR 1), typically 3 days after injury (IQR 6). The median length of hospital stay was 6 days (IQR 13), with an ICU stay of 1 day (IQR 5). In-hospital mortality was 10.4% and the median time to wound healing was 40 days (IQR 48).

**Conclusions:**

Creating an unfunded multicenter trial group for older adult burn patients is feasible. The group successfully conducted a retrospective study on care trends for burn-injured older adults. Key factors in the success of this initiative included standardized data collection protocols and strong collaboration between centers.

**Applicability of Research to Practice:**

Although challenges remain, this project illustrates the potential for establishing a larger, sustainable multicenter trial group aimed at improving clinical practices and outcomes for older burn patients.

**Funding for the Study:**

No financial compensation or technical support was provided to the participating institutions.

